# Morphological Analysis, Protein Profiling and Expression Analysis of Auxin Homeostasis Genes of Roots of Two Contrasting Cultivars of Rice Provide Inputs on Mechanisms Involved in Rice Adaptation towards Salinity Stress

**DOI:** 10.3390/plants10081544

**Published:** 2021-07-28

**Authors:** Shivani Saini, Navdeep Kaur, Deeksha Marothia, Baldev Singh, Varinder Singh, Pascal Gantet, Pratap Kumar Pati

**Affiliations:** 1 Department of Biotechnology, Guru Nanak Dev University, Amritsar 143005, Punjab, India; shivani.saini23@gmail.com (S.S.); kaurnavdeep302@yahoo.com (N.K.); deekshamarothia@yahoo.in (D.M.); bsbt89@gmail.com (B.S.); varinder75@gmail.com (V.S.); 2 Université de Montpellier, UMR DIADE, Centre de Recherche de l’IRD, Avenue Agropolis, BP 64501, CEDEX 5, 34394 Montpellier, France; 3 Centre of the Region Haná for Biotechnological and Agricultural Research, Department of Molecular Biology, Palacký University Olomouc, Šlechtitelů 27, 783 71 Olomouc, Czech Republic

**Keywords:** rice, abiotic stress, salinity, root, auxin, *YUCCA*, *PIN*, proteomics, mass spectrometry

## Abstract

Plants remodel their root architecture in response to a salinity stress stimulus. This process is regulated by an array of factors including phytohormones, particularly auxin. In the present study, in order to better understand the mechanisms involved in salinity stress adaptation in rice, we compared two contrasting rice cultivars—Luna Suvarna, a salt tolerant, and IR64, a salt sensitive cultivar. Phenotypic investigations suggested that Luna Suvarna in comparison with IR64 presented stress adaptive root traits which correlated with a higher accumulation of auxin in its roots. The expression level investigation of auxin signaling pathway genes revealed an increase in several auxin homeostasis genes transcript levels in Luna Suvarna compared with IR64 under salinity stress. Furthermore, protein profiling showed 18 proteins that were differentially regulated between the roots of two cultivars, and some of them were salinity stress responsive proteins found exclusively in the proteome of Luna Suvarna roots, revealing the critical role of these proteins in imparting salinity stress tolerance. This included proteins related to the salt overly sensitive pathway, root growth, the reactive oxygen species scavenging system, and abscisic acid activation. Taken together, our results highlight that Luna Suvarna involves a combination of morphological and molecular traits of the root system that could prime the plant to better tolerate salinity stress.

## 1. Introduction

The plant root is the vital organ that serves a wide range of functions and regulates crop productivity. As roots are in direct interface with the soil, they act as the primary site for perceiving environmental stress-related signals for plants [1,2]. Among various environmental stresses, salinity has emerged as one of the most serious threats limiting global crop production and yield [3]. Currently, almost 20% of the world’s total irrigated land is estimated to be affected by salinity stress and it is expected that by the end of the year 2050, more than 50% of the world’s arable land will become saline [4,5,6,7]. High soil salinity induces undesirable changes at phenotypic, biochemical, physiological, cellular, genetic and molecular levels, which are detrimental to plant growth and survival [8]. The root system responds to abiotic stresses by triggering stress adaptive mechanisms, which are supposed to be regulated by a number of factors [2,9,10].

The potential of several phytohormones to ameliorate the damaging effects of salinity stress has attracted the attention of researchers in the recent past [11,12]. Among different phytohormones, auxin is an important plant hormone well-known for controlling the different aspects of plant growth and development including tropistic growth, vascular tissue differentiation, auxiliary bud formation, cell elongation, flower organ development and abiotic stress tolerance [13,14,15,16]. It has also been regarded as a master player in triggering salinity stress-induced changes in root system architecture [12]. Auxin regulates the root growth rates by promoting lateral root formation and mediating the size of root meristem by controlling the transition from cell division to cell differentiation processes [17,18]. The processes that determine the spatiotemporal distribution of auxin and the maintenance of auxin homeostasis required for root growth and development include local auxin biosynthesis, transport, perception, signaling, conjugation and degradation [19,20].

Although roots are the critical site for the perception of salinity stress signals and are responsible for triggering stress-related mechanisms in plants, very little attention has been paid to analyzing this underground part of the plant in the context of understanding salinity tolerance. Physiological, biochemical and genetic studies have provided ample evidence in support of the key role of auxin in triggering abiotic stress-mediated differential modifications in the root system architecture of plants [21]. The key role of the maintenance of auxin homeostasis in regulating salinity stress tolerance is emerging in plant biology [22]. In the present study, in order to better understand the mechanisms conferring rice adaptation to salinity stress, we conducted a comparative analysis of various auxin-related genes (which regulate auxin homeostasis) in the roots of salt sensitive IR64 and salt tolerant Luna Suvarna (LS) cultivars of rice under optimal as well as salinity stress conditions. Further, the endogenous content of indole-3-acetic acid (IAA) has also been estimated in the roots of two contrasting salinity stress cultivars of rice, and an analysis of their root morphology has been performed.

For finding significant clues on the adaptive behavior of plants to salinity stresses, studies at the protein level might be a better option compared to at the transcript level since many post-transcriptional and post-translational changes often take place in plants and hence, the rate of transcription and the translation will not necessarily always correlate. Hence, proteome-based approaches involving two-dimensional gel electrophoresis (2-DE) and mass spectrometry (MS) are often utilized for unraveling proteins associated with induced changes in plants as they are very reliable, sensitive and powerful technologies [9,23]. For example, a comparative study of the leaf proteome profiles of the wild salt tolerant Poaceae species *Porteresia coarctata* with two rice cultivars variable in salt sensitivity—IR64 (salt-sensitive) and Pokkali (salt-tolerant)—suggested that, in the leaves of *Porteresia coarctata*, several proteins exhibited up-regulation that could provide it a physiological advantage under salinity stress [24]. However, there are limited reports on a comparative proteome analysis of the root of contrasting salt-responsive cultivars of rice. Therefore, herein a comparison of the root proteome of two rice cultivars differing in salt tolerance has been conducted using 2-DE and MS. Our results showed that salt tolerant rice cultivar LS has better stress adaptive root traits, elevated expression of auxin homeostasis genes and more endogenous IAA content than IR64 cultivar, which could be linked to the acquisition of natural salinity stress tolerance in LS. Further, several salinity stress responsive proteins were detected exclusively in the roots of LS, which might be providing a peculiar property for attaining salinity stress adaptation and tolerance in rice.

## 2. Results

### 2.1. Analysis of Morphological Parameters and IAA Quantification in IR64 and LS

**Figure 1:**
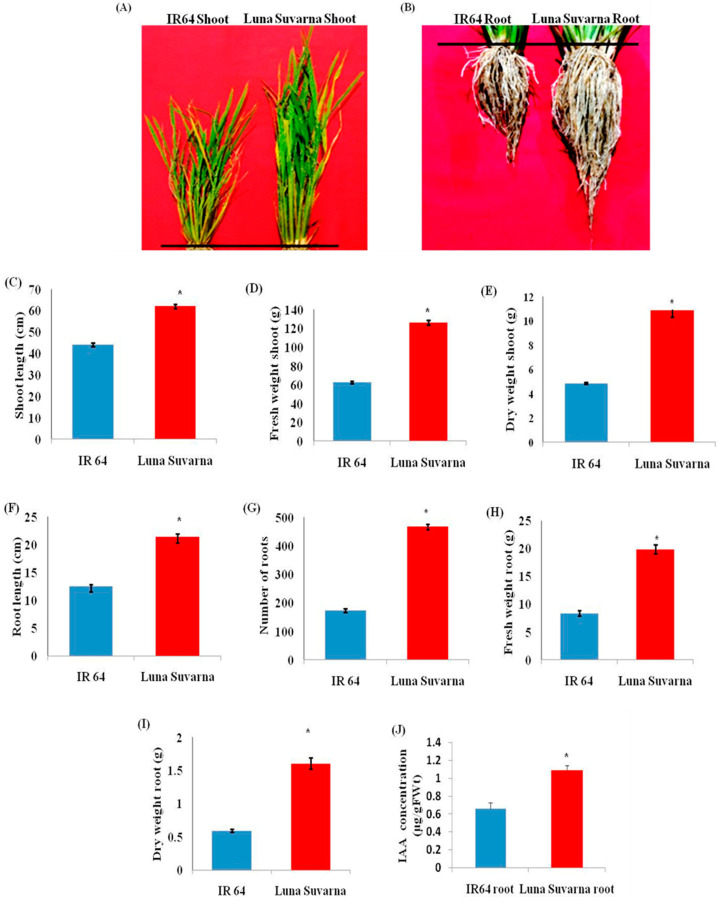
(**A**) Growth response of IR64 and Luna Suvarna (LS) shoot under control conditions; (**B**) Growth response of IR64 and Luna Suvarna (LS) roots under control conditions; (**C**) Comparative analysis of shoot length in IR64 and Luna Suvarna; (**D**) Comparative analysis of the fresh weight of shoot in IR64 and Luna Suvarna; (**E**) Comparative analysis of the dry weight of shoot in IR64 and Luna Suvarna; (**F**) Comparative analysis of root length in IR64 and Luna Suvarna; (**G**) Comparative analysis of the number of roots in IR64 and Luna Suvarna; (**H**) Comparative analysis of the fresh weight of roots in IR64 and Luna Suvarna; (**I**) Comparative analysis of the dry weight of roots in IR64 and Luna Suvarna; (**J**) IAA estimation in the roots of IR64 and LS. Data represent mean ± SE (n = 15) for the analysis of growth parameters while for IAA quantification mean ± SE (n = 3). Asterisks signs (*) represent values which were significantly different among different samples (Fisher LSD, *p* ≤ 0.05). Blue color represents IR64 while red color represents LS cultivar.

The differences in the morphological parameters of the two cultivars were clearly observable when cultivated in normal conditions (Figure 1A,B). An approximately 40% increase in the length of shoot and a 70% longer roots were observed in the salt-tolerant cultivar, LS, as compared to the sensitive cultivar IR64 (Figure 1A–C,F). The number of roots (primary root and crown roots) was also found to be 169% more in LS (Figure 1G). Moreover, an increase in the fresh weight of shoots and roots by 101% and 137% respectively, was noticed in LS (Figure 1D,H). Similarly, an approximately 122% and 170% enhancement in the dry weight of shoots and roots in the tolerant cultivar, respectively, was observed in LS with respect to IR64 (Figure 1E,I). The amount of endogenous IAA was also quantified in the roots of IR64 and LS. It was observed that in LS roots, the endogenous IAA concentration was significantly higher (1.086 µg/gFW) as compared to IR64 roots (0.6608 µg/gFW) (Figure 1J).

### 2.2. Expression Analysis of Genes Involved in Auxin Homeostasis in IR64 and LS Roots

**Figure 2:**
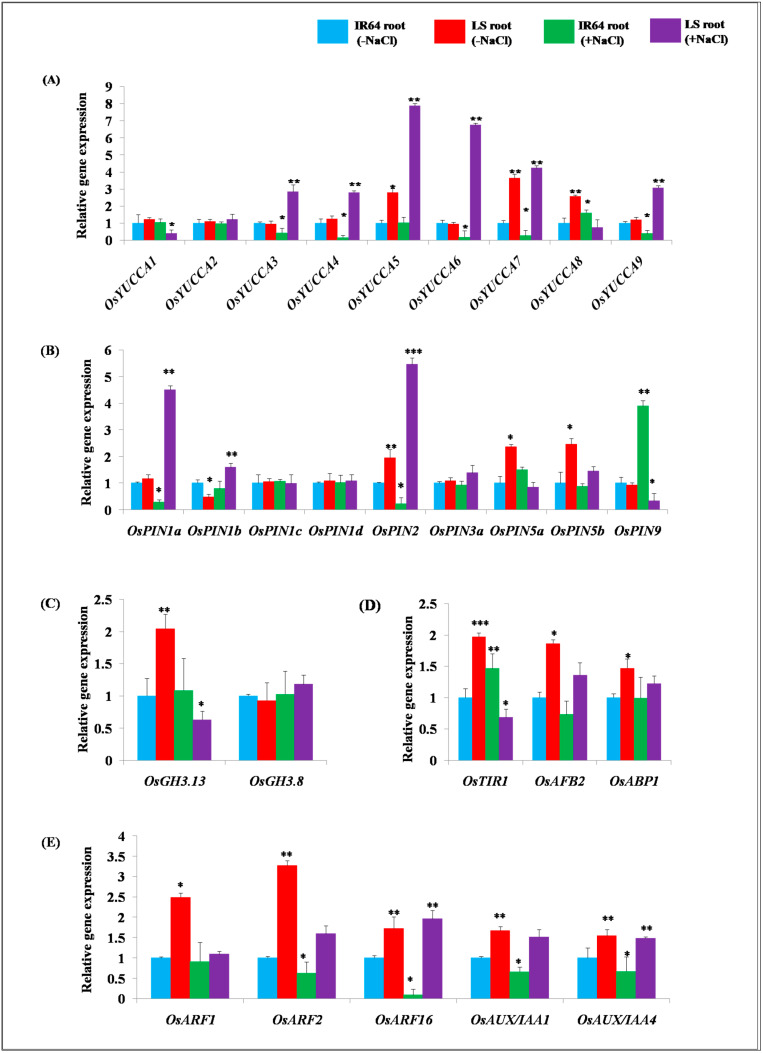
Real-time gene expression analysis of auxin homeostasis genes under control and salinity stress conditions in IR64 and LS roots. (**A**) Real-time gene expression of auxin biosynthesis genes; (**B**) Real-time gene expression of auxin transport genes; (**C**) Real-time gene expression of auxin conjugation and degradation genes; (**D**) Real-time gene expression of auxin receptor genes; (**E**) Real-time gene expression of auxin signaling genes. Three biological replicates were taken and bars represent mean ± SE. Asterisks signs (*, **, ***) represent values which were significantly different among different samples (Fisher LSD, *p* ≤ 0.05). The transcript levels of LS under normal condition and, LS and IR64 upon salinity stress treatment were compared with IR64 (control), whose expression was assumed as 1. (-NaCl) refers to untreated samples, (+NaCl) refers to salinity treated samples. Blue color refers IR64 root (-NaCl), red color refers to LS root (-NaCl), green color refers to IR64 root (+NaCl), and purple color refers to LS root (+NaCl).

To better understand the cause of the differences observed in the IAA content in the roots of both cultivars, the transcript levels of various genes involved in auxin homeostasis were measured by qRT-PCR under optimal and salinity stress conditions. Among various auxin biosynthesis genes, the transcript levels of *OsYUCCA5*, *OsYUCCA7*, and *OsYUCCA8* exhibited significant up-regulation of 2.79, 3.53, and 2.58 fold, respectively, in the roots of the salt-tolerant cultivar LS as compared to the salt-sensitive cultivar IR64 of rice under normal conditions (Figure 2A). In response to salinity stress, significant down-regulation of *OsYUCCA3, OsYUCCA4, OsYUCCA6, OsYUCCA7*, and *OsYUCCA9* by 0.43, 0.15, 0.18, 0.27, and 0.39 fold was observed in IR64 with respect to the control (Figure 2A). In the LS cultivar, auxin biosynthesis genes *OsYUCCA3, OsYUCCA4, OsYUCCA5, OsYUCCA6, OsYUCCA7,* and *OsYUCCA9* exhibited significant up-regulation by 2.83, 2.79, 7.88, 6.75, 4.53 and 3.07 fold, respectively, in the roots upon salinity stress with respect to the IR64 (control) (Figure 2A). On the contrary, the expression level of *OsYUCCA1* and *OsYUCCA8* exhibited significant down-regulation by 0.41 and 0.75 fold, respectively, in the LS root compared to the control (Figure 2A). The transcript levels of *OsYUCCA2* did not show any significant difference in two contrasting salinity stress responsive cultivars of rice under normal and salinity stress conditions.

Among different auxin efflux transporter *OsPIN* genes, *OsPIN2, OsPIN5a,* and *OsPIN5b* exhibited higher transcript level accumulation of 1.95, 2.36, and 2.46 fold, respectively, in the roots of untreated LS as compared to the IR64 (Figure 2B). On the contrary, significant down-regulation in the expression of *OsPIN1b*, that is, 0.48, was found in the roots of LS as compared to the salt-sensitive cultivar IR64 of rice (Figure 2B). In response to salinity stress in the IR64 cultivar, significant down-regulation of *OsPIN1a* and *OsPIN2* by 0.28 and 0.23 fold, respectively, was observed as compared to the control. The expression of auxin efflux transporters *OsPIN1a, OsPIN1b, OsPIN2, OsPIN3a,* and *OsPIN5b* was up-regulated by 4.51, 1.62, 5.46, 1.42, and 1.54 fold, respectively, in the roots of the LS cultivar under salinity stress as compared to the untreated IR64 control (Figure 2B). However, the transcript levels of *OsPIN1c* and *OsPIN1d* did not show any significant differences between the two cultivars in response to control or salinity stress conditions (Figure 2B). Under the effect of salinity stress, *OsPIN5a* and *OsPIN9* exhibited down-regulation by 0.85 and 0.33 fold, respectively, in LS than the roots of the control (Figure 2B).

The transcript levels of auxin conjugation and degradation gene *OsGH3.13* were found to be higher by 2.04 fold in the LS root under normal conditions (Figure 2C). In response to the salinity stress, no significant change was observed in the gene expression of *OsGH3.13* in IR64, whereas *OsGH3.13* displayed significant down-regulation by 0.63 fold in the LS root with respect to the control (Figure 2C). The gene expression of *OsGH3.8* did not exhibit a significant change in the roots of LS under the normal conditions with respect to IR64. *OsGH3.8* displayed 1.21 fold higher accumulations in the tolerant cultivar LS, and its expression remained unaltered in IR64 in response to salinity stress (Figure 2C). The expression of auxin receptor genes, particularly *OsTIR1, OsAFB2* and *OsABP1*, was studied in the roots of salt-sensitive cultivar IR64 and salt-tolerant cultivar LS of rice under normal and salinity stress (Figure 2D). It was observed that the expression of *OsTIR1* and *OsAFB2* displayed up-regulation by 1.96 and 1.86 fold in the roots of the LS under the normal conditions as compared to IR64. Similarly, the transcript level accumulation of *OsABP1*, the auxin receptor of the proteasome independent pathway was also found to be higher by 1.46 fold in the roots of LS than IR64 (Figure 2D). Under the effect of salinity stress, *OsTIR1* exhibited 1.46 fold up-regulation, while *OsAFB2* displayed 0.73 fold down-regulation in IR64 than in the control (Figure 2D). In LS roots, it was observed that the expression of *OsAFB2* and *OsABP1* displayed up-regulation by 1.36 and 1.22 fold, respectively, in response to salinity stress. On the contrary, *OsTIR1* showed 0.68 fold down-regulation in the roots of LS (Figure 2D). Interestingly, various auxin signaling genes, such as *OsARF1, OsARF2, OsARF16, OsAUX*/*IAA1* and *OsAUX*/*IAA4*, also exhibited higher gene expression of 2.49, 3.26, 1.72, 1.66, and 1.54 fold, respectively, in the LS root as compared to the IR64 under control conditions (Figure 2E). In response to salinity stress, the transcript levels of *OsARF1, OsARF2, OsARF16, OsAUX*/*IAA1*, and *OsAUX*/*IAA4* displayed down-regulation by 0.91, 0.62, 0.09, 0.6, and 0.67 fold, respectively, in IR64 with respect to the control. On the contrary, *OsARF2, OsARF16, OsAUX*/*IAA1* and *OsAUX*/*IAA4* exhibited up-regulation by 1.59, 1.96, 1.52, and 1.48 fold, respectively, in the LS root under salinity stress stimuli (Figure 2E). The expression level of *OsARF1* did not show any significant differences in the two contrasting salt tolerant cultivars of rice under salinity stress (Figure 2E).

### 2.3. 2-DE Analysis of Root Proteins in IR64 and LS

**Figure 3:**
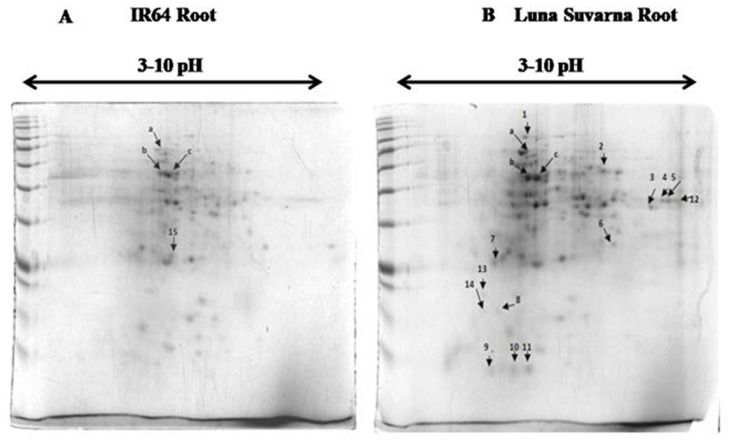
Two-dimensional gel electrophoretic analysis of the protein profiles of rice root proteome under control condition (**A**) IR64 root (**B**) Luna Suvarna (LS) root.

In complement to auxin-related gene expression analysis, the proteins isolated from roots of 14-days old seedlings of salt-sensitive IR64 and salt-tolerant LS cultivars of rice were subjected to 2-DE analysis. A total of 146 spots were detected in IR64 while 166 spots were observed in LS (Figure 3), using PDQuest 8.0.2 software. Among these protein spots, 27 were observed to be either present/absent and few were of altered intensity in the roots of IR64 and LS. These spots were excised and sent for MALDI TOF/TOF MS/MS analysis. Out of 27 spots, only 18 proteins were successfully identified (Table 1 and Table 2). The plant intracellular Ras group related LRR protein 2, B3 domain-containing protein, and Ubiquitin fold modifier protein 1 displayed higher protein expression by 1.76, 1.1, and 3.75 fold in the roots of LS in comparison to IR64, respectively (Table 2). Among the 18 identified proteins, 13 proteins were implicated in abiotic stress responses and two of them exhibited enzymatic activity. One protein constituted the core nucleosome component, however, another protein was involved in autophagy and protein transport and the remaining one protein function in the DNA methylation process.

**Figure 4:**
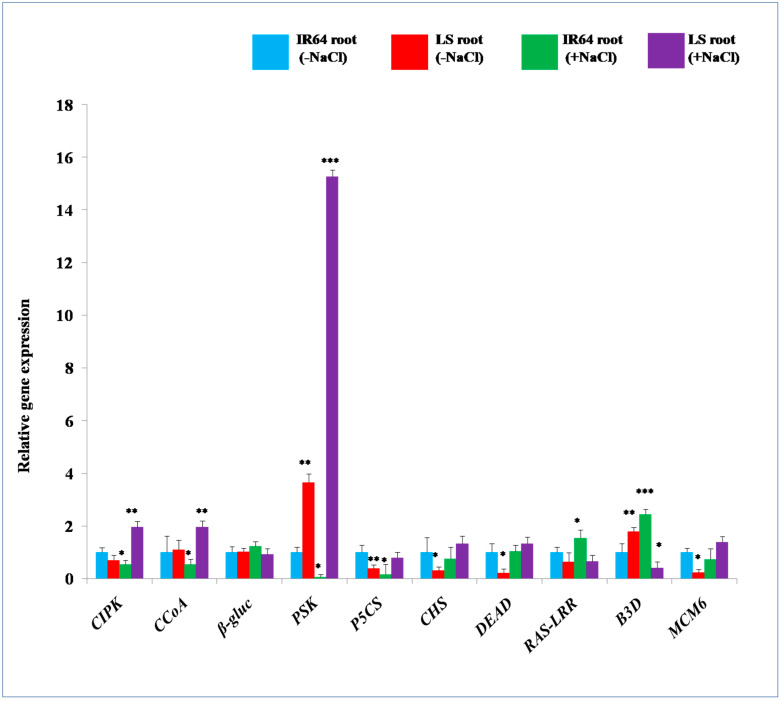
The relative expression levels of genes encoding for proteins differentially accumulated in IR64 and Luna Suvarna (LS) roots under control and salinity stress conditions. Three biological replicates were taken and bars represent mean ± SE. Asterisks signs (*, **, ***) represent values which were significantly different among different samples (Fisher LSD, *p* ≤ 0.05). The transcript levels of LS under normal condition and, LS and IR64 upon salinity stress treatment were compared with IR64 (control), whose expression was assumed as 1. (-NaCl) refers to untreated samples, (+NaCl) refers to salinity treated samples. Blue color refers to IR64 root (-NaCl), red color refers to LS root (-NaCl), green color refers to IR64 root (+NaCl) and purple color refers to LS root (+NaCl). CIPK: Calcineurin B-like interacting protein kinase 21; CCoA: 4-coumarate CoA ligase like 9; β-gluc: Beta-glucosidase 12; PSK: Phytosulfokines 3; P5CS: Delta-1-pyrroline-5-carboxylate synthase; CHS: Cyanate hydratase; DEAD: DEAD-box ATP-dependent RNA helicase 53; RAS-LRR: Plant intracellular Ras group related LRR protein 2; B3D: B3 domain-containing protein; MCM6: minichromosome maintenance 6.

Comparative expression analysis of identified stress marker genes (which are known for exhibiting tolerance against salinity stress) (Table 3) was performed in the roots of IR64 and LS under the native condition to complete the results obtained with MS at the transcript level. The transcript levels of *4-coumarate CoA ligase 9 (CCoA), β-glucosidases 12 (β-gluc), phytosulfokines 3 (PSK)*, and *B3 domain-containing protein (B3D)* were observed to be 1.09, 1.02, 3.64, and 1.78 fold higher, respectively, in LS roots compared to IR64 under normal conditions (Figure 4). However, the gene expression of *calcineurin B-like interacting protein kinase 21 (CIPK), delta-pyrroline-5-carboxylate synthase (P5CS), cyanate hydratase (CHS), DEAD-box ATP-dependent RNA helicases 53 (DEAD), plant intracellular Ras group related LRR protein 2 (RAS-LRR)*, and *minichromosome maintenance 6 (MCM6)* was 0.69, 0.38, 0.30, 0.21, 0.64, and 0.22 fold lower, respectively, in the roots of tolerant cultivar LS (Figure 4) with respect to the salt-sensitive IR64. Under salinity stress, *β-gluc* and *B3D* displayed up-regulation of 1.22 and 2.44 fold, respectively, in IR64 roots compared to the control (Figure 4). On the contrary, *CIPK, CCoA, PSK, P5CS, CHS,* and *MCM6* showed lower transcript levels by 0.54, 0.53, 0.066, 0.15, 0.75, and 0.72 fold, respectively, in IR64, while *DEAD* exhibited no significant change in its expression. In response to salinity stress, the expression of *CIPK, CCoA, PSK, CHS, DEAD* and *MCM6* was 1.85, 1.86, 15.26, 1.32, 1.5, and 1.37 fold higher respectively, in LS roots compared to control IR64 (Figure 4). However, the transcript level of *β-gluc, P5CS, B3D* and *RAS-LRR* exhibited 0.83, 0.78, 0.41, and 0.65 fold (Figure 4) lower transcript level accumulation, respectively, in the roots of the tolerant cultivar (LS) under salinity stress stimuli.

## 3. Discussion

Plant roots perceive the salinity stress signals and promptly pass them to the shoot to activate various stress-responsive pathways [1,2,9,10]. Although roots are the important site for the perception of salinity stress-related signals, not much attention has been paid to exploring this underground part of the plant in the context of understanding salinity tolerance attributes. In the present study, a positive correlation has been observed between the root system architecture, auxin content, stress marker proteins, and salinity stress adaptation. It was observed that the salinity stress tolerant LS cultivar of rice has a longer primary root, a larger number of roots and a higher fresh weight and dry weight in comparison to IR64. It is realized that plants acquire deeper roots, more lateral roots, more root hair length, and a larger number of roots and its biomass for achieving natural defense against stress conditions including salinity [25,26,27]. Moreover, it has been demonstrated that the presence of a larger root/shoot length ratio and a higher root biomass promoted the adaptation of plants towards environmental stresses [24,28]. Thus, the present observation of distinct differences in the root phenotype of LS compared to IR64 could be extrapolated to the acquisition of adaptive morphological traits that enable LS plants to mitigate salinity stress when exposed.

It is believed that phytohormones are critical signaling molecules that function downstream of environmental stimuli and regulate various stress adaptive pathways [29]. In previous studies, it has been demonstrated that high salinity stress greatly affects root architecture by inhibiting primary and lateral root growth through altering the accumulation and distribution of the critical phytohormone, auxin [30,31,32,33]. Among different auxins, the role of primary auxin IAA is thought to be fundamental as it is the key player in regulating root development [34,35]. Thus, in the present work, the endogenous levels of IAA have been estimated. Upon analysis, it was observed that LS exhibited significantly higher IAA content compared to IR64 roots. Earlier, it was reported that *iaam-OX* transgenic lines (with higher endogenous IAA level) and wild-type plants of Arabidopsis pretreated with IAA exhibited resistance towards drought stress [15]. However, the triple mutants, *yuc1yuc2yuc6,* which were deficient in endogenous IAA content, showed decreased resistance towards drought stress [15]. Moreover, augmented levels of indole-3-butyric acid (IBA) in growing leaves and higher IAA content in the roots of the highly salt-resistant maize variety, SR03, were observed in response to salinity stress [32]. It was revealed that the increased IAA concentration enhanced the accumulation of cell growth-related agents, such as β-expansins (involved in cell wall extension), under salinity stress [32,36]. In Arabidopsis *iar4* mutants, reduced root meristem activity and root growth were reported due to diminished auxin distribution in root tips, indicating the key role of auxin in root growth and development [30]. The exogenous application of auxin is also well known to positively modulate root architecture, especially the lateral root number [15,33,37,38]. Thus, the higher endogenous levels of IAA observed in salt tolerant rice cultivar (LS) could be considered as one of the prominent reasons for the acquisition of salinity stress adaptive root traits observed in the LS cultivar.

It is well realized that the process of auxin-mediated root development is regulated by a complex interplay between auxin metabolism, its signaling and transport leading to the spatio-temporal distribution of auxin [12,39,40]. Thus, to get insights into the molecular dynamics of auxin homeostasis, the transcript-level expression of different genes involved in the auxin pathway has been analyzed in roots in the present work. Recent studies suggest that the local biosynthesis of auxin by YUCCA flavin monooxygenases in the roots is the primary source for normal root development and root gravitropic responses [35]. Moreover, it has been demonstrated that five *YUCCA* genes—*YUCCA3*, *YUCCA5*, *YUCCA7*, *YUCCA8*, and *YUCCA9*—express highly in Arabidopsis roots, playing an essential role in the root development [35]. However, the link of *YUCCA* genes and salinity stress adaptation has never been evaluated in rice. Interestingly, in the present study, it was observed that the transcript level accumulation of different *OsYUCCA* genes was higher in the roots of LS. It might be the primary reason for more auxin biosynthesis and its accumulation in LS roots. Further, in response to salinity stress, the transcript level accumulation of *OsYUCCA3, OsYUCCA4, OsYUCCA5, OsYUCCA6, OsYUCCA7,* and *OsYUCCA9* was enhanced in LS roots. In Arabidopsis, *YUCCA8* and *YUCCA9* have been linked with the development of lateral roots, while their mutants develop shorter primary roots suggesting their key role in the development of root system architecture [41]. Hence, the present study hints towards a link between *OsYUCCA* genes mediated enhanced auxin accumulation and subsequently better developed root system architecture for the acquisition of salinity stress tolerance in rice.

Once IAA is biosynthesized, it is transported to the area of its requirement with the help of cell-to-cell auxin transport mediated by *OsPINs* in rice [42]. Earlier, it was found that polar auxin transport is affected by osmotic stress caused by increased salinity or drought [31]. Moreover, flavonoids and phenolic compounds that are accumulated in response to stress exposure also inhibit polar auxin transport [43,44]. Interestingly, in the present study, the transcript levels of auxin efflux carrier genes, such as *OsPIN1a, OsPIN2, OsPIN3a*, *OsPIN5a*, and *OsPIN5b*, were found to be higher in the LS root. Moreover, under salinity stress, the expression of auxin transport genes, particularly *OsPIN1a, OsPIN1b, OsPIN2, OsPIN3a,* and *OsPIN5b*, was up-regulated in the roots of LS. *OsPIN1b* and *OsPIN9* have been suggested to participate in root development in rice, by regulating auxin-cytokinin interaction [45]. Further, *OsPIN2* expresses highly in roots and enhances shoot to root auxin transport [46]. Thus, the increased expression of such transporter genes in LS roots suggests that salt-tolerant rice cultivar has better capability to maintain auxin homeostasis under salinity stress; however, further investigations are necessary to consolidate these findings.

The optimum concentration of IAA is maintained in a cell through their conjugation and degradation by *OsGH3* genes [47]. Hence, the expression of auxin conjugation and degradation gene *OsGH3.13* was analyzed in the roots of salt-sensitive (IR64) and salt-tolerant cultivar (LS) of rice. It was observed that the expression of *OsGH3.13* was significantly higher in the roots of LS, which is contradictory to the observed higher IAA content in LS. Thus, it can be inferred that the role of the *OsYUCCA* genes is probably more critical in regulating auxin content in rice as compared to *OsGH3*. Further, the analysis of *OsGH3.8* at transcript level suggests no difference in expression level between IR64 and LS roots. However, under salinity stress, the transcript level accumulation of *OsGH3.13* was down-regulated in the roots of LS compared to IR64. On the contrary, *OsGH3.8* exhibited up-regulation in the root but not to a significant level. It indicates that the lower expression of *OsGH3.13* under NaCl application in LS root might be responsible for providing tolerance against salinity stress, probably by enhancing IAA levels.

Various findings have suggested that *OsAFB2* and *OsTIR1* are the auxin signaling receptors affected by salinity stress [31,48]. However, their probable role in providing a natural defense against salinity stress has never been evaluated. In the present study, the transcript level accumulation of auxin receptor genes *OsTIR1, OsAFB2,* and *OsABP1* were found to be elevated in the roots of LS with respect to IR64 under normal conditions. This identification of the enhanced expression of auxin receptors can be linked to higher auxin content in the roots of LS compared to IR64. Under salinity stress, the expression of *OsTIR1* showed down-regulation in LS roots with respect to control IR64. On the contrary, the transcript levels of *OsAFB2* and *OsABP1* exhibited up-regulation in the roots of LS compared to IR64 under the salt application. The expression of various auxin signaling genes, *OsARF1, OsARF2, OsARF16, OsAUX*/*IAA1*, and *OsAUX*/*IAA4*, was also found to be higher in the roots of LS compared to IR64 under both control as well as salinity stress conditions, demonstrating elevated endogenous IAA level in LS root compared to IR64. Previous studies have also linked elevated auxin concentration with increased auxin transport and downstream signaling genes [49], thus promoting auxin-mediated root development. In rice, 31 auxin repressor (*OsAUX*/*IAAs*) and 25 auxin activator (*OsARFs)* genes that participate in auxin signaling were observed to be suppressed by cold, heat and drought stress [31]. On the contrary, some *OsAUX*/*IAA,* such as *OsAUX*/*IAA 6,9,18,19,20* and *28* and *OsARF 4,11,13,14,16,18* and *19,* were induced by at least one among cold, heat, and drought stress [31]. Hence, various auxin signaling genes respond differentially to abiotic stresses such as cold, heat and drought [31]. However, to the best of our knowledge, there is no report on the effect of salinity stress on the auxin signaling genes. It has been suggested that, in Arabidopsis Aux/IAA protein, IAA14 participates in the early stages of lateral root development [50,51]. Hence, the observed elevated expression of *AUX*/*IAA* in LS root compared to IR64 might be extrapolated to the salt tolerance and a higher number of roots detected in the tolerant cultivar.

The comparative proteomics study revealed that some proteins were specifically present in the roots of LS compared to IR64. Among these, CIPK21 and CIPK29, the Ca^2+^ sensing proteins of the CIPK gene family, have been previously linked with the enhanced tolerance against salinity stress conditions in Arabidopsis [52,53]. It was suggested that *salt overly sensitive 3* (*SOS3*) encodes for CBL, which functions in sensing the cytosolic Ca^2+^ concentration by directly binding to it [52,54]. The Ca^2+^ bound CBL proteins directly activate their interacting partners, such as CIPK6, which are involved in auxin transport, regulation of root length and lateral root development [52,54]. CIPK6 also enhances the transcript levels of *NAC, PIN2,* and *P5CS* genes, which promote salinity stress resistance in plants [52]. P5CS protein, which is involved in proline biosynthesis, was also observed to show increased levels in LS roots in the current study. Proline is involved in the maintenance of cell turgor or osmotic balance, stabilizing membranes to prevent the leakage of electrolytes, and regulates reactive oxygen species (ROS) homeostasis [55,56]. Thus, it can be supposed that the increased levels of an enzyme involved in proline biosynthesis could be responsible for enhancing the proline content in the LS roots that has been previously linked to salinity tolerance [2]. In LS roots, a higher abundance of lignin biosynthesis protein, CCoA, was also observed. There is evidence that salinity stress causes increased lignification of the cell wall through maintaining the structural rigidity and durability of desert poplar plants [57]. Thus, the current finding indicates the possible role of lignin deposition in enhancing salinity stress tolerance in rice [57,58,59]. Further, higher protein accumulation of detoxification enzyme, CHS (which detoxifies cytotoxic compounds such as cyanate), was observed exclusively in LS roots. CHS also supplies salinity-stressed plants with alternative sources of nitrogen and carbon for better adaptation [60,61]. PSK, which was found exclusively in LS roots, is linked to plant immunity and the maintenance of cellular homeostasis, and is also involved in normal root growth and development [62]. PSK also decreases ethylene production which hinders the primary root growth by inhibiting cell proliferation in the meristematic zone and cell elongation in the elongation zone [62,63,64]. Hence, the augmented root growth and salinity stress tolerance observed in LS compared to IR64 could also be linked to the higher PSK content. Another stress marker protein, DEAD, was observed exclusively in the roots of LS. In earlier reports, DEAD has been shown to provide salinity stress tolerance in transgenic tobacco by reducing oxidative stress through activating the ROS scavenging system [65,66]. It also improves a plant’s photosynthesis machinery, enhances plant growth and development, and mitigates salinity stress [65]. Hence, the higher accumulation of DEAD in LS roots might be involved in scavenging excess ROS, leading to the promotion of salinity stress tolerance. Moreover, β-gluc, which enhances the ABA pool, was also found exclusively in LS roots. The key role of β-gluc in releasing active and free forms of abscisic acid (ABA) from physiologically inactive ABA-glucose conjugate pool, resulting in the alleviation of salinity stress, has already been reported [67,68]. Therefore, the higher accumulation of β-gluc in LS roots might promote ABA accumulation, thus enhancing salinity stress tolerance. The content of MCM6 protein was found exclusively in the roots of the salt tolerant cultivar (LS), which plays an important role in the initiation and elongation steps of eukaryotic DNA replication [69]. In one of the previous studies, the role of MCM6 in providing resistance against high salinity and cold stress has already been elucidated [69]. It was also suggested that the ectopic over-expression of *Pisum sativum PsMCM6* in tobacco confers salinity stress tolerance without affecting yield [65,69]. Further, there were some proteins, such as RAS-LRR, B3D and ubiquitin fold modifier 1, that expressed relatively higher in LS as compared to IR64 roots. The enhanced protein accumulation of RAS-LRR (which encodes polygalacturonase inhibitor proteins, PGIPs) plays a critical role in mitigating salinity stress [68,70]. Further, the roots of LS also exhibited higher protein accumulation of B3D (which triggers various stress-responsive genes) with respect to IR64 roots. RAV (related to ABI3/VP1) protein contains AP2 domain at N-terminal region and B3D in its C-terminal region, which also confer salinity stress resistance through regulating various stress-related genes (*RD29A, RD29B, RAB18, ABI1, ERD15, KIN, ERD10,* and *COR15a*) [71]. The protein content of ubiquitin fold modifier 1 (UFM1) was found to be several-fold higher in the roots of the tolerant cultivar (LS), which could prevent oxidative damage caused by free radicals. It has been suggested that, in addition to ubiquitin, plants utilize a number of ubiquitin-like proteins, such as those related to ubiquitin 1 (RUB1), small ubiquitin-like modifier (SUMO), UFM1, and homology to ubiquitin (HUB), which participates in providing abiotic stress tolerance [72,73]. These proteins confer resistance against salinity stress by prohibiting the damage caused by free radicals and also prevent endoplasmic reticulum-induced apoptosis in protein secretory cells [73,74]. In IR64, upon the application of salinity stress, the expression of *CIPK*, *CCoA, P5CS, PSK, CHS,* and *MCM6* genes exhibited down-regulations with respect to the control IR64, which might lead to salinity stress susceptibility in the sensitive cultivar. The present finding also indicates that, in IR64, the protein turnover rate might be high, probably leading to targeting of the salinity stress responsive proteins towards degradation, leading to salinity stress sensitivity. On the contrary, in LS upon salinity stress application, higher transcript accumulation of *CIPK*, *CCoA, PSK, CHS, DEAD* and *MCM6* was observed, which could be linked to its acquisition of the salinity stress resistance property.

**Table 3:** Role of identified stress marker proteins in salinity stress tolerance.

Protein	Functions	Reference
Calcineurin B-like interacting protein kinases	(1) Enhances shoot to root auxin transport. (2) Mediates root development and lateral root formation.(3) Promotes salt stress tolerance.	[52,53,54]
4-Coumarate CoA ligase	(1) Enhances salt stress tolerance. (2) Increases lignification of salt stress-tolerant varieties.	[57,58]
β-glucosidases	(1) In vacuoles, it converts ABA glucopyranosides into free ABA. (2) It leads to the adaptation of plants to salt stress conditions.	[67,68]
Phytosulfokines	(1) Emerged as a novel kind of plant hormone recently and involved in immunity and homeostasis. (2) Involved in root growth and development; and inhibits ethylene production.	[62,63,64]
Plant intracellular Ras-group-related LRR protein 2	(1) Promotes root development. (2) Promotes salt stress tolerance by encoding PGIPs.	[68,70]
Minichromosome maintenance 6 (MCM6)	(1) It confers salinity stress tolerance in pea by additional uptake of Na^+^	[65,69]
B3 Domain containing protein	(1) RAV (Related to ABI3 and VP1) has AP2 and B3 domain. (2) Promotes salinity tolerance, enhances stress marker genes.	[71]
Cyanate hydratase	(1) Involved in detoxification of cyanate. (2) Provide alternative sources of nitrogen and carbon for enhancing salt stress tolerance.	[60,61]
Delta pyrroline-5-carboxylate synthase	(1) Maintenance of cell turgor, osmotic balance and lipid synthesis. (2) Salt stress resistance.	[55,56]
DEAD-box ATP-dependent RNA helicase 53 (DEAD)	(1) ReduceS oxidative stress through activation of ROS scavenging system. (2) Improves plant’s photosynthesis machinery, enhancing plant growth and development and mitigates salt stress.	[65,66]

## 4. Materials and Methods

### 4.1. Plant Material

The certified and disease-free seeds of salinity stress-sensitive IR64 and salinity stress-tolerant LS rice (*Oryza sativa* L.) cultivars were procured from Punjab Agricultural University, Ludhiana, India, and the Central Rice Research Institute (CRRI), Cuttack, India, respectively. LS can tolerate the salt stress up to 8 dSm^−1^. The seeds were surface sterilized with 70% ethanol (*v*/*v*) for 1 min and treated with 0.4% sodium hypochlorite solution containing a drop of tween-20 for 30 min. The seeds were washed thrice with autoclaved distilled water and were then dried on autoclaved Whatman paper (3 mm) for 5 min. After surface sterilization, the seeds were inoculated in the plastic tray containing autoclaved sand moistened with sterile distilled water and were incubated in the culture room at 25 °C (day/night), 70–80% relative humidity (day/night), and 14 h photoperiod for 14 days. After 14 days, IR64 and LS seedlings were treated with 100 mM NaCl for 8 h for imposing salinity stress. The roots were later separated for protein and RNA extraction to conduct 2-dimensional gel electrophoresis and gene expression studies, respectively.

### 4.2. Study of Morphological Parameters

The seedlings of IR64 and LS were harvested after 2 months and were dipped in water to remove the adhering sand particles. A representative sample of 15 seedlings of both IR64 and LS were selected to study the morphological parameters. Root and shoot length were measured using a meter scale and observations for fresh weight were taken in grams. The root and shoot of each sample were then dried in an oven at 70 °C until a constant weight was achieved, and then the observations for dry weight were recorded. The number of roots for each seedling of IR64 and LS was also counted.

### 4.3. IAA Estimation

To estimate the content of IAA, 5 g fresh roots of IR64 and LS were crushed finely in liquid nitrogen and extracted in chilled 80% ethanol (15 mL/g) containing butylated hydroxytoluene (BHT) (100 mg/L) [75]. The homogenized material was kept in the dark at 4 °C for 24 h and was filtered. The solid residues were re-extracted thrice with 80% ethanol for 4 h without adding BHT. The BHT containing filtrate and the filtrate without BHT were combined and were centrifuged at 8000 rpm for 20 min. The supernatant was concentrated by drying at 30 °C in a rotavapor in the dark and was used for further processing while the pellet was discarded. The concentrate was resuspended in 2.5 mL of 0.1 M potassium phosphate buffer (pH 8) and was applied to the PVP column after adding 3-bed volumes of potassium phosphate buffer into the PVP column. After elution, a 3-bed volume of potassium phosphate buffer in the PVP column was added again. The elute was concentrated by drying in the rotavapor at 30 °C to obtain 10 mL of elute and its pH was adjusted to 2.5 with 1N HCl. The concentrated 10 mL elute was dissolved in diethyl ether (30 mL) containing BHT (100 mg/L). It was vortexed and kept for 10 min and then the supernatant was taken in a fresh flask (approx. 30 mL). This step was repeated four times. The obtained elute was mixed with 1.5 g of Na_2_SO_4_ and kept for 30 min. After 30 min, it was evaporated and dried completely at 30 °C using the rotavapor. Then 5 mL of distilled water was added and evaporated on rotavapor at 30 °C. The step was repeated twice and a dried pellet was obtained. The pellet was then dissolved in 1.5 mL of methanol (HPLC grade) for IAA estimation. Further, the elution was carried out with 100 % methanol (HPLC grade): Water (Formic acid 0.05% *v*/*v*), 35:65, at a flow rate of 1 mL·min^−1^. The column elutes were passed through a UV detector at 254 nm, and IAA was estimated with reference to an authentic standard of IAA (1 mM) (Sigma Chemical Co., St. Louis, MO, USA). The readings were taken in the replicates of three and the average of peaks was obtained.

### 4.4. RNA Extraction and cDNA Synthesis

A total of 150 mg of root sample of IR64 and LS was homogenized in liquid nitrogen using pestle and mortar. Total RNA was isolated using Trizol reagent (Invitrogen, http://www.invitrogen.com, last accessed on 20 July 2021), as per the manufacturer’s instructions. RNase-free DNase (Sigma-Aldrich, USA) was used to remove the genomic DNA and 2 µg of RNA was used to synthesize cDNA in a total volume of 10 µL reaction using the iScript cDNA synthesis kit (Bio-Rad, Hercules, CA, USA) as per the manufacturer’s recommendations.

### 4.5. Quantitative Real-Time (qRT) PCR Analysis

qRT-PCR was performed to study the differential expression of auxin homeostasis genes in the roots of IR64 and LS. The nucleotide sequences of different genes involved in auxin homeostasis were retrieved from the rice annotation project database (RAP-DB) and the gene-specific primers were designed using Integrated DNA Technologies, USA (http://www.idtdna.com/primerquest/Home/Index, last accessed on 20 July 2021) (Supporting Information-Table S1). The qRT-PCR reaction was performed in 96 well plates using SYBR Green detection chemistry in the StepOne Plus Realtime PCR machine (Applied Biosystems, Waltham, MA, USA). A 10 µL reaction was prepared using 5 µL of 2X Fast SYBR Green (Applied Biosystem), 7.5 ng of each cDNA, 5 μmol each of forward and reverse gene-specific primers and the final volume was raised to 10 µL using sterile nuclease-free water. No template control (NTC) was also set for each primer pair. The thermal cycling was carried out using the following parameters: initial denaturation step at 95 °C for the 20 s to activate the Taq DNA polymerase, followed by the 40 cycles of denaturation at 95 °C for 3 s and finally annealing at 60 °C for 30 s. The melting curve was generated by heating the amplicon from 60 to 90 °C. Baseline and threshold cycles (Ct) were automatically determined using the StepOne Plus Software version 2.3 (Applied Biosystems, USA). Fold changes were calculated using C_T_ (∆∆C_T_) and normalized against *OsUBQ5* (LOC_Os1g328400) used as an endogenous control.

### 4.6. Protein Extraction

A phenol-based method was used for extracting proteins from 1 g roots of IR64 and LS as reported previously [76]. The samples were homogenized with 6 mL of extraction buffer containing 100 mM KCl, 700 mM sucrose, 50 mM EDTA, and 500 mM Tris-HCl pH 8.0. Further, 2% β-mercaptoethanol, 1 mM PMSF, and a 10 mM protease inhibitor cocktail were added to the extraction buffer just before use. The mixture was vortexed and incubated by agitating on ice for 10 min. After incubation, 6 mL of tris-buffered phenol was added to it and the mixture was again vortexed and incubated on a shaker on ice for 10 min. The homogeneous mixture was centrifuged at 12,000 rpm at 4 °C for 20 min. The upper phenolic phase was collected carefully in a fresh tube. Again, 3 mL of the extraction buffer was added to the tris-buffered phenol and the extraction process was repeated and the upper phenolic phase was collected. Further, 5 volumes of 0.1 M ammonium acetate in 100% cold methanol were added to the phenolic phase and the tube was shaken gently. The mixture was incubated at −20 °C for protein precipitation overnight. The protein pellet was recovered after 24 h by centrifugation at 12,000 rpm at 4 °C for 10 min and the supernatant was discarded. The pellet so obtained was washed thrice with 0.1 M ammonium acetate in cold methanol and then with a mixture containing 80% methanol and 20% acetone, followed by washing with 100% cold methanol. The final washing was given with 100% chilled acetone and the washed pellet was air-dried and stored at −80 °C for 2-DE.

### 4.7. Protein Solubilization and Quantification

The protein pellets were suspended thoroughly in rehydration buffer (ReadyPrep™ 2-D Starter Kit Rehydration/Sample Buffer #1632106, Bio-Rad, USA). Protein concentration was quantified with a Bradford protein estimation assay [77] using bovine serum albumin (BSA) taken as standard.

### 4.8. Two-Dimensional Gel Electrophoretic (2-DE) Analysis

For isoelectric focusing (IEF), 150 µg of protein was dissolved in a total of 130 µL of rehydration buffer containing 8 M urea, 2% CHAPS, 50 mM DTT, 0.2% Bio-Lyte^®^ 3/10 ampholyte, 0.001% bromophenol blue (Bio-Rad, USA) and passively rehydrated over IPG strips (7 cm, pH 3–10, Readystrips, Cat. No. 163-200, Bio-Rad, USA) overnight at 20 °C. After rehydration, the strips were focused at 250V for 40 min, 4000 V for 2 h with linear voltage amplification, and finally to 10,000 V h with rapid amplification. After IEF, the strips were incubated with equilibration buffer I, containing 6 M urea, 375 mM Tris-HCL pH 8.8, 2% SDS and 2% DTT for 15 min for reduction (ReadyPrep 2-D Starter Kit Equilibration Buffer I #1632107, Bio-Rad USA). For alkylation of the proteins, the strip was further incubated with 2.5% iodoacetamide dissolved in equilibration buffer II containing 6 M urea, 375 mM Tris-HCL pH 8.8 and 2% SDS (ReadyPrep 2-D Starter Kit Equilibration Buffer I #1632108) for 15 min. The second-dimensional electrophoresis was performed using 12% polyacrylamide gel. After mounting the strip on the gel, it was sealed with 0.5% agarose containing 0.1% bromophenol blue, and the protein molecular marker was also loaded. Electrophoresis was performed at a constant voltage of 100 V for 2 h in tris-glycine-SDS containing running buffer.

### 4.9. Gel Staining, Imaging, and Analysis

After 2-DE, gels were stained with Coomassie brilliant blue and were stored in 5% acetic acid until further analysis. Gel imaging was conducted using the Molecular Imager Gel Doc XR system (Bio-Rad, USA) and the images were analyzed using PDQuest 8.0.2 software.

### 4.10. Protein in-Gel Digestion and Mass Spectrometry (MS) Analysis

Proteins spots showing variations in their intensities, presence and absence were manually excised from Coomassie brilliant blue-stained gels and were subjected to mass spectrometric analysis [78]. The excised gel pieces were destained properly using 100 mM NH_4_HCO_3_/50% ACN solution and washed twice with 200 µL of Milli-Q water for 5 min each and were dehydrated using 100 µL of acetonitrile. The samples were subjected to trypsinolysis in 25 µL of trypsin solution (Sigma, USA) with a concentration of 20 µg/mL in 25 mmol/L NH_4_HCO_3,_ and were incubated overnight at 37 °C. Each digested peptide was further extracted from the gels using 50% trifluoroacetic acid/ 50% acetonitrile, twice at room temperature. The extracted peptides were mixed with 0.5 µL of α-cyano-4-hydroxy-cinnamic acid (Bruker) of a concentration of 20 mg/mL prepared in 0.1% trifluoroacetic acid, 30% (*v*/*v*) acetonitrile and dried at room temperature. The trypsin digested protein samples were subjected to mass spectrometric analysis using an UltrafleXtreme™ mass spectrometer (Bruker Daltonics Inc. Germany). The instrument was calibrated and fine-tuned with a mass standard starter kit (Bruker) and standard tryptic digested BSA (Bruker, Germany). TOF spectra were recorded in positive ion reflector mode between mass ranges of 700–3500 Da. For protein characterization, the obtained MS spectra were searched against a non-redundant database (SwissProt database) using a MASCOT search engine with these parameters: taxonomy: *Oryza sativa* (rice); parent ion mass tolerance was set at ± 1.2 Da and MS/MS tolerance at 100ppm; variable modifications, oxidation of methionine (M) and carbadomethylation of cysteine (C) and trypsin enzyme.

### 4.11. Statistical Analysis

All the data obtained from different experiments were evaluated using statistical analysis. An unpaired t-test and a one-way analysis of variance (ANOVA) (the Fischer LSD, Waltham, MA, USA) test were conducted to compare the mean differences using Sigma Stat version 3.5. Comparisons with *p* < 0.05 were considered significantly different.

## 5. Conclusions

The present study shows that salt tolerant rice cultivars present salinity stress adaptive root traits, likely due to an elevated endogenous auxin content and augmented levels of key salinity stress providing proteins in its roots. Salt tolerant rice LS cultivars exhibited higher transcript-level expression of different genes involved in auxin homeostasis both under control and salinity stress conditions. Thus, our study suggests that an elevated level of auxin and a higher buffering capacity of the auxin homeostasis process may be critical for the acquisition of salinity stress adaptation in rice. Upon 2-DE and MS analysis, several salinity stress tolerance providing proteins were detected that exhibited higher constitutive expression in the roots of LS with respect to IR64. In LS roots, the transcript level of some identified stress marker proteins exhibited lower expression; on the contrary, their protein accumulation was higher in the tolerant cultivar, LS. It indicates that their protein turnover rate might be low. Taken together; these results highlight morphological and molecular features that are critical for rice adaptation towards salinity stress and reveal that this process is multifactorial. Moreover, our results pinpoint several candidate genes that could be artificially overexpressed to increase salinity stress tolerance in rice.

## Figures and Tables

**Table 1:** Specifically expressed proteins in Luna Suvarna (LS) versus IR64 roots identified using MALDI-ToF/ToF mass spectrometry.

Spot	Protein Name	Accession Number	MSU Number	Reference Organism	Mrpi (Theoretical)	Score	Coverage	Function
1	Histone H2B.10	H2B10_ORYSI	LOC_Os01g05610.1	*Oryza sativa* subsp. Indica	16522 (10.02)	41	36%	Core nucleosome component
2	DEAD-box ATP-dependent RNA helicase 53	RH53_ORYSJ	LOC_Os07g05050.1	*Oryza sativa* subsp. Japonica	65429 (9.44)	47	15%	Abiotic stress responses
3	Glyceraldehyde-3-phosphate dehydrogenase 3 cytosolic	G3PC3_ORYSJ	LOC_Os02g38920	*Oryza sativa* subsp. Japonica	36716 (7.68)	37	38%	Glycolysis enzyme
4	Calcineurin B-like interacting protein kinase 21	CIPKL_ORYSJ	LOC_Os07g44290	*Oryza sativa* subsp. Japonica	59592 (9.26)	39	15%	Abiotic stress tolerance
5	4-Coumarate-CoA ligase-like 9	4CLL9_ORYSJ	LOC_Os04g24530	*Oryza sativa* subsp. Japonica	59782 (5.69)	46	22%	Abiotic stress tolerance
6	β-glucosidases 12	BGL12_ORYSJ	LOC_Os04g39880	*Oryza sativa* subsp. Japonica	57713 (8.75)	47	11%	Abiotic stress tolerance
7	Ubiquitin-like protein autophagy-related	ATG12_ORYSI	LOC_Os06g10340	*Oryza sativa* subsp. Indica	10454/9.05	34	32%	Autophagy and protein transport
8	Phytosulfokines 3	PSK3_ORYSJ	LOC_Os03g47230	*Oryza sativa* subsp. Japonica	8341/5.83	34	29%	Abiotic stress tolerance
9	Cyanate hydratase	CYNS_ORYSI	LOC_Os10g33270	*Oryza sativa* subsp. Indica	18653/5.61	33	44%	Abiotic stress tolerance
10	Probable protein arginine N-methyltransferase	ANM3_ORYSI	LOC_Os07g44640	*Oryza sativa* subsp. Indica	69127/4.49	29	13%	Mediates methylation process
11	Heat stress transcription factor B 1	HSFB1_ORYSJ	LOC_Os09g28354	*Oryza sativa* subsp. Japonica	33121/9.35	33	43%	Abiotic stress responses
12	Delta 1-pyrroline-5-carboxylate synthase	P5CS_ORYSJ	LOC_Os05g38150	*Oryza sativa* subsp. Japonica	78153/6.37	53	14%	Abiotic stress tolerance
13	Calcineurin B-like-interacting protein kinase 29	CIPKT_ORYSJ	LOC_Os07g48090	*Oryza sativa* subsp. Japonica	48581/8.69	38	44%	Abiotic stress tolerance
14	Minichromosome maintenance 6	MCM6_ORYSI	LOC_Os05g14590	*Oryza sativa* subsp. Indica	93168/5.55	43	17%	Abiotic stress tolerance
15	UDP-glucose 6-dehydrogenase 1	UGDH1_ORYSJ	LOC_Os03g31210	*Oryza sativa* subsp. Japonica	52834 (5.75)	39	13%	Enzymatic function

Spots 1–14 were exclusively observed in the case of Luna Suvarna root, whereas spot 15 was differentially less expressed in the IR64 root.

**Table 2:** Highly expressed proteins in Luna Suvarna (LS) roots as compared to IR64 identified using MALDI-ToF/ToF mass spectrometry.

Spot	Protein Name	Accession Number	MSU Number	Reference Organism	Mrpi (Theoretical)	Mrpi (Experimental)	Score	Coverage	Function
a	Plant intracellular Ras group related LRR protein 2	PIRL2_ORYSJ	LOC_Os02g38040	*Oryza sativa* subsp. Japonica	55345/5.51	114.7/5.6	30	27%	Abiotic stress tolerant
b	Ubiquitin-fold modifier 1	UFM1_ORYSJ	LOC_Os01g73140	*Oryza sativa* subsp. Japonica	10356/9.60	81.67/5.98	30	44%	Abiotic stress tolerant
c	B3 domain-containing protein	Y1237_ORYSJ	LOC_Os01g52540	*Oryza sativa* subsp. Japonica	83749/5.81	118.37/5.71	38	34%	Abiotic stress tolerant

## Data Availability

The available data are presented in the manuscript.

## Supplementary Materials

The following are available online at https://www.mdpi.com/article/10.3390/plants10081544/s1, Table S1: List of primers used for the quantitative real-time polymerase chain reaction.

## References

[1] Jackson M. (1997). Hormones from roots as signals for the shoots of stressed plants. Trends Plant Sci..

[2] Saini S., Kaur N., Pati P.K. (2018). Reactive oxygen species dynamics in roots of salt sensitive and salt tolerant cultivars of rice. Anal. Biochem..

[3] Gerona M.E.B., Deocampo M.P., Egdane J.A., Ismail A.M., Dionisio-Sese M.L. (2019). Physiological Responses of Contrasting Rice Genotypes to Salt Stress at Reproductive Stage. Rice Sci..

[4] Qadir M., Quillérou E., Nangia V., Murtaza G., Singh M., Thomas R.J., Drechsel P., Noble A.D. (2014). Economics of salt-induced land degradation and restoration. Nat. Resour. Forum..

[5] Shrivastava P., Kumar R. (2015). Soil salinity: A serious environmental issue and plant growth promoting bacteria as one of the tools for its alleviation. Saudi. J. Biol. Sci..

[6] Egamberdieva D., Wirth S., Bellingrath-Kimura S.D., Mishra J., Naveen K., Arora N.K. (2019). Salt-Tolerant Plant Growth Promoting Rhizobacteria for Enhancing Crop Productivity of Saline Soils. Front. Microbiol..

[7] Solis C.A., Yong M.T., Vinarao R., Jena K., Holford P., Shabala L., Zhou M., Shabala S., Chen Z. (2020). Back to the Wild: On a Quest for Donors toward Salinity Tolerant Rice. Front. Plant Sci..

[8] AbdElgawad H., Zinta G., Hegab M.M., Pandey R., Asard H., Abuelsoud W. (2016). High salinity induces different oxidative stress and antioxidant responses in maize seedlings organs. Front. Plant Sci..

[9] Zhao Q., Zhang H., Wang T., Chen S., Dai S. (2013). Proteomics-based investigation of salt-responsive mechanisms in plant roots. J. Proteomics.

[10] Ghosh D., Xu J. (2014). Abiotic stress responses in plant roots: A proteomics perspective. Front. Plant Sci..

[11] Ryu H., Cho Y. (2015). Plant hormones in salt stress tolerance. J. Plant Biol..

[12] Korver R.A., Koevoets I.T., Testerink C. (2018). Out of Shape during Stress: A Key Role for Auxin. Trends Plant Sci..

[13] Quint M., Gray W.M. (2006). Auxin signaling. Curr. Opin. Plant Biol..

[14] Davies P.J., Davies P.J. (2010). Plant hormones: Their nature, occurrence, and functions. Plant Hormones.

[15] Shi H., Chen L., Ye T., Liu X., Ding K., Chan Z. (2014). Modulation of auxin content in Arabidopsis confers improved drought stress resistance. Plant Physiol. Biochem..

[16] Jadamba C., Kang K., Paek N., Lee S.I., Yoo S. (2020). Overexpression of Rice *Expansin7* (*Osexpa7*) Confers Enhanced Tolerance to Salt Stress in Rice. Int. J. Mol. Sci..

[17] Fukaki H., Okushima Y., Tasaka M. (2007). Auxin-mediated lateral root formation in higher plants. Int. Rev. Cytol..

[18] Di Mambro R., De Ruvo M., Pacifici E., Salvi E., Sozzani R., Benfey P.N., Busch W., Novak O., Ljung K., Di Paola L. (2017). Auxin minimum triggers the developmental switch from cell division to cell differentiation in the Arabidopsis root. Proc. Natl. Acad. Sci. USA.

[19] Rosquete M.R., Barbez E., Kleine-Vehn J. (2012). Cellular auxin homeostasis: Gatekeeping is housekeeping. Mol. Plant.

[20] Saini S., Sharma I., Pati P.K., Pandey G.K. (2017). Integrating the Knowledge of Auxin Homeostasis with Stress Tolerance in Plants. Mechanism of Plant Hormone Signaling under Stress.

[21] Ribba T., Garrido-Vargas F., O’Brien J.A. (2020). Auxin-mediated responses under salt stress: From developmental regulation to biotechnological applications. J. Exp. Bot..

[22] Vaseva I.I., Mishev K., Depaepe T., Vassileva V., Van Der Straeten D. (2021). The Diverse Salt-Stress Response of Arabidopsis ctr1-1 and ein2-1 Ethylene Signaling Mutants is Linked to Altered Root Auxin Homeostasis. Plants.

[23] Zhang H., Han B., Wang T., Chen S., Li H., Zhang Y., Dai S. (2012). Mechanisms of plant salt response: Insights from proteomics. J. Proteome Res..

[24] Sengupta S., Majumder A.L. (2009). Insight into the salt tolerance factors of a wild halophytic rice, *Porteresia coarctata*: A physiological and proteomic approach. Planta.

[25] Comas L.H., Becker S.R., Cruz V.M.V., Byrne P.F., Dierig D.A. (2013). Root traits contributing to plant productivity under drought. Front. Plant Sci..

[26] Kim Y., Chung Y.S., Lee E., Tripathi P., Heo S., Kim K.H. (2020). Root response to drought stress in rice (*Oryza sativa* L.). Int. J. Mol. Sci..

[27] Arif M.R., Islam M.T., Robin A.H.K. (2019). Salinity stress alters root morphology and root hair traits in *Brassica napus*. Plants.

[28] Polania J., Poschenrieder C., Rao I., Beebe S. (2017). Root traits and their potential links to plant ideotypes to improve drought resistance in common bean. Theor. Exp. Plant Physiol..

[29] Ku Y., Sintaha M., Cheung M., Lam H. (2018). Plant Hormone Signaling Crosstalks between Biotic and Abiotic Stress Responses. Int. J. Mol. Sci..

[30] Fu Y., Yang Y., Chen S., Ning N., Hu H. (2019). Arabidopsis IAR4 modulates primary root growth under salt stress through ROS-mediated modulation of auxin distribution. Front. Plant Sci..

[31] Du H., Liu H., Xiong L. (2013). Endogenous auxin and jasmonic acid levels are differentially modulated by abiotic stresses in rice. Front. Plant Sci..

[32] Zörb C., Geilfus C.M., Mühling K.H., Ludwig-Müller J. (2013). The influence of salt stress on ABA and auxin concentrations in two maize cultivars differing in salt resistance. J. Plant Physiol..

[33] Koevoets I.T., Venema J.H., Elzenga J.T.M., Testerink C. (2016). Roots Withstanding their Environment: Exploiting Root System Architecture Responses to Abiotic Stress to Improve Crop Tolerance. Front. Plant Sci..

[34] Saini S., Sharma I., Kaur N., Pati P.K. (2013). Auxin: A master regulator in plant root development. Plant. Cell Rep..

[35] Chen Q., Dai X., De-Paoli H., Cheng Y., Takebayashi Y., Kasahara H., Kamiya Y., Zhao Y. (2014). Auxin overproduction in shoots cannot rescue auxin deficiencies in Arabidopsis roots. Plant. Cell Physiol..

[36] Kao C. (2017). Mechanisms of Salt Tolerance in Rice Plants: Cell Wall-Related Genes and Expansins. J. Taiwan Agric. Res..

[37] Cheng Y., Dai X., Zhao Y. (2006). Auxin biosynthesis by the YUCCA flavin monooxygenases controls the formation of floral organs and vascular tissues in Arabidopsis. Genes Dev..

[38] Alarcón M., Salguero J., Lloret P.G. (2019). Auxin modulated initiation of lateral roots is linked to pericycle cell length in maize. Front. Plant Sci..

[39] Ljung K. (2013). Auxin metabolism and homeostasis during plant development. Development.

[40] Fukui K., Hayashi K.I. (2018). Manipulation and sensing of auxin metabolism, transport and signaling. Plant Cell Physiol..

[41] Hentrich M., Böttcher C., Düchting P., Cheng Y., Zhao Y., Berkowitz O., Masle J., Medina J., Pollmann S. (2013). The jasmonic acid signaling pathway is linked to auxin homeostasis through the modulation of YUCCA8 and YUCCA9 gene expression. Plant J..

[42] Zwiewka M., Bilanovičová V., Seifu Y.W., Nodzyński T. (2019). The Nuts and Bolts of PIN Auxin Efflux Carriers. Front. Plant Sci..

[43] Bielach A., Hrtyan M., Tognetti V.B. (2017). Plants under stress: Involvement of auxin and cytokinin. Int. J. Mol. Sci..

[44] Potters G., Pasternak T.P., Guisez Y., Jansen M.A. (2009). Different stresses, similar morphogenic responses: Integrating a plethora of pathways. Plant Cell Environ..

[45] Singh P., Mohanta T.K., Sinha A.K. (2015). Unraveling the Intricate Nexus of Molecular Mechanisms Governing Rice Root Development: OsMPK3/6 and Auxin-Cytokinin Interplay. PLoS ONE.

[46] Balzan S., Johal G.S., Carraro N. (2014). The role of auxin transporters in monocots development. Front. Plant Sci..

[47] Kong W., Zhong H., Deng X., Gautam M., Gong Z., Zhang Y., Zhao G., Liu C., Li Y. (2019). Evolutionary Analysis of *GH3* Genes in Six *Oryza* Species/Subspecies and Their Expression Under Salinity Stress in *Oryza sativa* ssp. Japonica. Plants.

[48] Xia K., Wang R., Ou X., Fang Z., Tian C., Duan J., Wang Y., Zhang M. (2012). OsTIR1 and OsAFB2 downregulation via OsmiR393 overexpression leads to more tillers, early flowering and lesstolerance to salt and drought in rice. PLoS ONE.

[49] Xu D., Miao J., Yumoto E., Yokota T., Asahina M., Watahiki M. (2017). YUCCA9 Mediated Auxin Biosynthesis and Polar Auxin Transport Synergistically Regulate Regeneration of Root Systems Following Root Cutting. Plant Cell Physiol..

[50] Guseman J.M., Hellmuth A., Lanctot A., Feldman T.P., Moss B.L., Klavins E., Calderón Villalobos L.I., Nemhauser J.L. (2015). Auxin-induced degradation dynamics set the pace for lateral root development. Development.

[51] Leyser O. (2018). Auxin Signaling. Plant Physiol..

[52] Tripathi V., Parasuraman B., Laxmi A., Chattopadhyay D. (2009). CIPK6, a CBL-interacting protein kinase is required for development and salt tolerance in plants. Plant J..

[53] Chen L., Wang Q.Q., Zhou L., Ren F., Li D.D., Li X.B. (2013). Arabidopsis CBL-interacting protein kinase (CIPK6) is involved in plant response to salt/osmotic stress and ABA. Mol. Biol. Rep..

[54] Hu W., Xia Z., Yan Y., Ding Z., Tie W., Wang L., Zou M., Wei Y., Lu C., Hou X. (2015). Genome-wide gene phylogeny of CIPK family in cassava and expression analysis of partial drought-induced genes. Front. Plant Sci..

[55] Hayat S., Hayat Q., Alyemeni M.N., Wani A.S., Pichtel J., Ahmad A. (2012). Role of proline under changing environments: A review. Plant Signal. Behav..

[56] Gharsallah C., Fakhfakh H., Grubb D., Gorsane F. (2016). Effect of salt stress on ion concentration, proline content, antioxidant enzyme activities and gene expression in tomato cultivars. AoB Plants.

[57] Zhang C.H., Ma T., Luo W.C., Xu J.M., Liu J.Q., Wan D.S. (2015). Identification of 4CL Genes in Desert Poplars and Their Changes in Expression in Response to Salt Stress. Genes.

[58] Shafi A., Chauhan R., Gill T., Swarnkar M.K., Sreenivasulu Y., Kumar S., Kumar N., Shankar R., Ahuja P.S., Singh A.K. (2015). Expression of SOD and APX genes positively regulates secondary cell wall biosynthesis and promotes plant growth and yield in Arabidopsis under salt stress. Plant Mol. Biol..

[59] Le Gall H., Philippe F., Domon J.M., Gillet F., Pelloux J., Rayon C. (2015). Cell Wall Metabolism in Response to Abiotic Stress. Plants.

[60] Nveawiah-Yoho P., Zhou J., Palmer M., Sauve R., Zhou S. (2013). Identification of Proteins for Salt Tolerance Using a Comparative Proteomics Analysis of Tomato Accessions with Contrasting Salt Tolerance. J. Am. Soc. Hortic. Sci..

[61] Maršálová L., Vítámvás P., Hynek R., Prášil I.T., Kosová K. (2016). Proteomic Response of *Hordeum vulgare* cv. Tadmor and *Hordeum marinum* to Salinity Stress: Similarities and Differences between a Glycophyte and a Halophyte. Front. Plant Sci..

[62] Lorbiecke R., Steffens M., Tomm J.M., Scholten S., Wiegen P., Kranz E., Wienand U., Sauter M. (2005). Phytosulphokine gene regulation during maize (*Zea mays* L.) reproduction. J. Exp. Bot..

[63] Sauter M. (2015). Phytosulfokine peptide signaling. J. Exp. Bot..

[64] Kutschmar A., Rzewuski G., Stührwohldt N., Beemster G.T., Inzé D., Sauter M. (2009). PSK-α promotes root growth in Arabidopsis. New Phytol..

[65] Macovei A., Vaid N., Tula S., Tuteja N. (2012). A new DEAD-box helicase ATP-binding protein (OsABP) from rice is responsive to abiotic stress. Plant Signal. Behav..

[66] Tuteja N., Banu M.S., Huda K.M., Gill S.S., Jain P., Pham X.H., Tuteja R. (2014). Pea p68, a DEAD-box helicase, provides salinity stress tolerance in transgenic tobacco by reducing oxidative stress and improving photosynthesis machinery. PLoS ONE.

[67] Dietz K.J., Sauter A., Wichert K., Messdaghi D., Hartung W. (2000). Extracellular beta-glucosidase activity in barley involved in the hydrolysis of ABA glucose conjugate in leaves. J. Exp. Bot..

[68] Li W., Zhao F., Fang W., Xie D., Hou J., Yang X., Zhao Y., Tang Z., Nie L., Lv S. (2015). Identification of early salt stress responsive proteins in seedling roots of upland cotton (*Gossypium hirsutum* L.) employing iTRAQ-based proteomic technique. Front. Plant Sci..

[69] Dang H.Q., Tran N.Q., Gill S.S., Tuteja R., Tuteja N. (2011). A single subunit MCM6 from pea promotes salinity stress tolerance without affecting yield. Plant Mol. Biol..

[70] Xu Z.S., Xiong T.F., Ni Z.Y., Chen. X.P., Chen M., Li L.C., Gao D.Y., Yu X.D., Liu P., Ma Y.Z. (2009). Isolation and identification of two genes encoding leucine-rich repeat (LRR) proteins differentially responsive to pathogen attack and salt stress in tobacco. Plant Sci..

[71] Li X.J., Li M., Zhou Y., Hu S., Hu R., Chen Y., Li X.B. (2015). Overexpression of cotton RAV1 gene in Arabidopsis confers transgenic plants high salinity and drought sensitivity. PLoS ONE.

[72] Miura K., Hasegawa P.M. (2010). Sumoylation and other ubiquitin-like post-translational modifications in plants. Trends Cell Biol..

[73] Srivastava A.K., Zhang C., Yates G., Bailey M., Brown A., Sadanandom A. (2016). SUMO is a Critical Regulator of Salt Stress Responses in Rice. Plant Physiol..

[74] Lemaire K., Moura R.F., Granvik M., Igoillo-Esteve M., Hohmeier H.E., Hendrickx N., Newgard C.B., Waelkens E., Cnop M., Schuit F. (2011). Ubiquitin fold modifier 1 (UFM1) and its target UFBP1 protect pancreatic beta cells from ER stress-induced apoptosis. PLoS ONE.

[75] Nagar P.K., Sood S. (2006). Changes in endogenous auxins during winter dormancy in tea (*Camellia sinensis* L.) O. Kuntze. Acta Physiol. Plant.

[76] Faurobert M., Pelpoir E., Chaïb J. (2007). Phenol extraction of proteins for proteomic studies of recalcitrant plant tissues. Methods Mol. Biol..

[77] Bradford M. (1976). A rapid and sensitive method for the quantitation of microgram quantities of protein utilizing the principle of protein-dye binding. Anal. Biochem..

[78] Kaur D., Dogra V., Thapa P., Bhattacharya A., Sood A., Sreenivasulu Y. (2015). In vitro flowering associated protein changes in *Dendrocalamus hamiltonii*. Proteomics.

