# Supplementary material for: Morphological Analysis, Protein Profiling and Expression Analysis of Auxin Homeostasis Genes of Roots of Two Contrasting Cultivars of Rice Provide Inputs on Mechanisms Involved in Rice Adaptation towards Salinity Stress
Source: Plants (Basel). 2021 Jul 28;10(8):1544. doi: 10.3390/plants10081544 (PMC8399380; doi:10.3390/plants10081544)
Supplement: Supplementary file 1 [file plants-10-01544-s001.zip › plants-1204819-supplementary.pdf]

Morphological analysis, protein profiling and expression analysis of auxin homeostasis genes of roots of two contrasting cultivars of rice provide inputs on mechanisms involved in rice adaptation towards salinity stress

Shivani Saini[1], Navdeep Kaur[1], Deeksha Marothia[1], Baldev Singh[1], Varinder Singh[1], Pascal Gantet[2,3]* and Pratap Kumar Pati[1]*

[1] Department of Biotechnology, Guru Nanak Dev University, Amritsar-143005, Punjab, India

[2] Université de Montpellier, UMR DIADE, Centre de Recherche de l'IRD, Avenue Agropolis, BP 64501, 34394, Montpellier cedex 5, France

[3]Department of Molecular Biology, Centre of the Region Haná for Biotechnological and Agricultural Research, Palacký University Olomouc, Šlechtitelů 27, Olomouc, 783 71, Czech Republic

***Correspondence**

Dr. Pratap Kumar Pati,

Department of Biotechnology, Guru Nanak Dev University, Amritsar-143005 (Punjab), India.

E-mail: pkpati@yahoo.com, Tel: +91-183-2255802-09 Ext No. 3177, Fax: +91-183-2258272.

***Co-Correspondence**

Dr. Pascal Gantet

[2] Université de Montpellier, UMR DIADE, Centre de Recherche de l'IRD, Avenue Agropolis, BP 64501, 34394, Montpellier cedex 5, France

[3]Department of Molecular Biology, Centre of the Region Haná for Biotechnological and Agricultural Research, Palacký University Olomouc, Šlechtitelů 27, Olomouc, 783 71, Czech Republic

E-mail:pascal.gantet@umontpellier.fr

Supplementary Material

Table S1. List of primers used for quantitative real time polymerase chain reaction.

**Gene**	**Locus ID**	**Forward primer**	**Reverse Primer**
OsYUCCA1	LOC_Os01g45760	5′-GGACGCTAGACCACATCAAATC-3′	5′-AATTGCTCCTCCTTGCCATC-3′
OsYUCCA2	LOC_Os05g452	5′-	5′-GTAACCCGTGGCGAGTATAATG-3′

	40	CATCAAGGTGTATCCGGAGTTG-3′	
OsYUCCA3	LOC_Os01g53200	5′-GATTGATGGCCTAGACGACTTC-3′	5′-CACTCCCAACAACGAGAACA-3′
OsYUCCA4	LOC_Os01g12490	5′-GAACCCTAGCCAAGATCAAGTC-3′	5′-AGTGCTGCCATCCACAAA-3′
OsYUCCA5	LOC_Os12g32750	5′-CCTCTCAGTGTTCCTTCTCAAG-3′	5′-CGGAGGCCGTATTTCTCAAT-3′
OsYUCCA6	LOC_Os07g25540	5′-TCATACTGGCCACCGGATA-3′	5′-TTCCAACCATCTGGGAATGG-3′
OsYUCCA7	LOC_Os04g03980	5′-CGACATCAACCCGCTCTT-3′	5′-GCTGCCAATGTACTCCATCT-3′
OsYUCCA8	LOC_Os04g03980	5′-GGCCCATTGGAGATCAAGAA-3′	5′-CTCCACCTCCGGTACTATCTT-3′
OsYUCCA9	LOC_Os01g16714.1	5′-GAATCTACGGCAGCTACGAG-3′	5′-AGCCATTGTTGTGGGTAGG-3′
OsPIN1a	LOC_Os02g50960	5′-GACCTCCACATGTTCGTCTG-3′	5′-GTCCTTCGCTCCATCCATTT-3′
OsPIN1b	LOC_Os11g64190	5′-GAATCGTGCCCTTTGTGTTTG-3′	5′-GAGCAATCAGCATCCCGAATA-3′
OsPIN1c	LOC_Os06g12610	5′-ATCACCCTCTTCTCCCTCTC-3′	5′-GATCTGCACCATGAGGCTAC-3′
OsPIN1d	LOC_Os12g04000	5′-CGGTCGATTTCGATCCTTTCA	5′-GCATACGAAGCAAGGGAGTT-3′
OsPIN2	LOC_Os06g44970	5′-AGGCATTGTCCCGTTTGT-3′	5′-GTAGAGTATCGTGATCGGAAGC-3′
OsPIN3a	LOC_Os01g45550	5′-ACACTTACTCCAGCCTCCT-3′	5′-GAGATGGACTTCTCGACGATTG-3′
OsPIN5a	LOC_Os01g69090	5′-CCCTACCTCAATCCATCACATC-3′	5′-GTAGGGAGACAAGCATTCCAA-3′
OsPIN5b	LOC_Os09g32770	5′-ACAGGTGGCATGTCGAAAC-3′	5′-CTGCAACGCCATGAACAAAC-3′
OsPIN9	LOC_Os01g58860	5′-AAGCTTCCTTGGCCTCATC-3′	5′-GGTACGAATGGTGAACAGAGAG-3′

OsGH3.8	LOC_Os07g40290.1	5′-CTCTACTTCCTGTTCGTCAAGTC-3′	5′-TTGAAGTGGTCGCTCTTGTAG
OsGH3.13	LOC_Os11g32510	5′-CACTACGTGCCACCTTGTCCTT-3′	5′-GTGTCCTCTTCTGGGA-3′
OsTIR1	LOC_Os05g05800	5′-GACCAGATGTCACAACCTGAA-3′	5′-CAACTAGCTGAGGAGCCTTATG-3′
OsAFB2	LOC_Os04g32460	5′-GGTTCAGGGCTCTTGTTCTTA-3′	5′-CCTCGATCCTCCACTTCATTT-3′
OsABP1	LOC_Os12g34460	5′-AAAGGGAAAGGGACACTCTTG-3′	5′-ACCTGGTGCGGATCATTTAC-3′
OsARF1	LOC_Os11g32110	5′-GGAAGATCCTGTGTGAGGTTATG-3′	5′-CTCCTCAGTAGAGCCGTTATCT-3′
OsARF2	LOC_Os01g70270	5′-CGAGCCCTTCAGAGTTCATTAT-3′	5′-TGCCTCTTCTCCTTCAAACC-3′
OsARF16	LOC_Os01g13520	5′-CGTCTGTTTGGTGTGGATTTG-3′	5′-TCGTGAGAACCAGTACCTTTG-3′
OsAUX/IAA1	LOC_Os01g08320	5′-CCCTGGAAGATGTTTGTGGA-3′	5′-GGCTCTTGGTGCTAAGTTGA-3′
OsAUX/IAA4	LOC_Os01g18360	5′-ATGTTCAAGACGCCCATCA-3′	5′-GTCTTCATAGGTGAGGACATGG-3′
CIPK21	LOC_Os07g44290	5′-CCGGAGAATGTCCTAGTTGATG-3′	5′-CACATGTGGTATGCAACAATCC-3′
CCoA9	Os04g0100075	5′-ACCTGGTGTCCAACCTCT-3′	5′-ATGTGGAAGAACGGCATGAG-3′
B-glu	LOC_Os04g39880	5′-TACCACAAGCACCTCCTTTC-3′	5′-CGTTCGACCACTCGAAGTTAT-3′
PSK	LOC_Os03g01008.1	5′-GGAAACTACGATGGTAGAGTGG-3′	5′-TAGTCCAGATGAGCCACGA-3′
P5CS	LOC_Os05g38150	5′-GGTACCGGAAGCTTGTCAATAG-3′	5′-GTAAAGAGCCATCAGTCCACTC-3′
CHS	LOC_Os10g33270.1	5′-TGAGGAGTTTGGTGATGGAATC-3′	5′-GACCACCACGCGATCTTT-3′
DEAD	LOC_Os07g05050	5′-CTGCTCGTGGATTAGACATACC-3′	5′-CACTTCCTTTCTTCCCTGCT-3′

RAS-LRR	LOC_Os02g38040	5′-AAGAATGTCCCAGATGCCATAG-3′	5′-TCTTGAGGCTGGTGAGGA-3′
B3D	LOC_Os01g52540	5′-GAGAAGTGTAGGCTAGTTGATGAG-3′	5′-CTTCGTGGAGTTTCAGGACTAC-3′
MCM6	LOC_Os05g14590.1	5′-GCTGGAGACACGGTCATATTTA-3′	5′-GGACACCAGACCCATTCTTT-3′
Ubiquitin5 (UBQ5)	LOC_Os1g328400	5′-CCTTCATGGCCAACCACTT-3′	5′-CTAAGCCTGCTGGTTGTAGAC-3′
